# Assessment of Mineral Nutrient Efficiency in Genetically Diverse Spinach Accessions by Biochemical and Functional Marker Strategies

**DOI:** 10.3389/fpls.2022.889604

**Published:** 2022-05-30

**Authors:** Madiha Rashid, Zubaida Yousaf, Ahmad Din, Muneeb Munawar, Arusa Aftab, Nadia Riaz, Afifa Younas, Ibrahim A. Alaraidh, Mohammad K. Okla, Hamada AbdElgawad

**Affiliations:** ^1^Department of Botany, Lahore College for Women University, Lahore, Pakistan; ^2^Department of Botany, Division of Science and Technology, University of Education, Lahore, Pakistan; ^3^Institute of Biological Sciences, University of Malaya, Kuala Lumpur, Malaysia; ^4^National Institute of Food Science and Technology, Faculty of Food, Nutrition and Home Sciences, University of Agriculture, Faisalabad, Pakistan; ^5^Vegetable Research Institute, Ayub Agricultural Research Institute, Faisalabad, Pakistan; ^6^Department of Botany and Microbiology, College of Science, King Saud University, Riyadh, Saudi Arabia; ^7^Integrated Molecular Plant Physiology Research Group, Department of Biology, University of Antwerp, Antwerp, Belgium

**Keywords:** accession, breeding, crop improvement, nitrate, mineral uptake, oxalate, spinach

## Abstract

Leafy vegetable crops are considered as a natural source of mineral nutrients that could decrease the risk factor of many growth issues in children and adults. Spinach is globally considered as the most desirable leafy crop, due to its taste and nutrient richness along with greater nitrate contents and better nitrogen use efficiency. To evaluate the mineral nutrient efficiency of this crop, thirty genetically diverse spinach accessions were analyzed through nutritional and functional marker strategies. The accession 163,310 from Pakistan was found to be rich in minerals (sodium, calcium, potassium, zinc, and manganese) and nitrates. However, the oxalate contents were lesser in the accessions that had greater quantity of nutrients. These represented a negative correlation between mineral availability and oxalate accumulation in the leaves. To study the relationship of oxalates and minerals in the accessions, a functional marker analysis was performed, based on the genes involved in oxalate metabolism and disease resistance in spinach. High level of genetic polymorphism was observed among the accessions represented with 115 polymorphic bands out of 130 bands. Heat map clustering represented the accessions from Asian countries (Pakistan, India, China, and Iran) as the most adaptable accessions to the local environment. The correlation between nutritional and genetic analysis also revealed the nutrient richness of these accessions along with good oxalate metabolism and disease resistance. Hence, these accessions could be considered as useful genotypes in future breeding programs.

## Introduction

Mineral nutrients play a vital role in proper growth and functioning of plants. A number of nutrients, including iron, magnesium, manganese, zinc, etc., act as cofactors of various enzymes, required for normal functioning of plant (Karthika et al., 2018). The deficiency of essential nutrients and vitamins, around the world, is leading toward high rate of physiological disorders, including slow growth and bones’ problem (Cousins, 2006; Abu Al-Qumboz and Abu-Naser, 2019). Therefore, a nutrient-rich food is needed as a supplement for the proper growth of both the children and adults. Leafy vegetables are a good source of nutrients that can potentially decrease the risk factor of these disorders (Odhav et al., 2007). Spinach (*Spinacia oleracea* L.) is a nutritionally rich leafy vegetable belonging to family Amaranthaceae. It is native to central Asia and thought to be originated from Iran (Sabaghnia et al., 2014). It is being cultivated in more than 60 countries. Moreover, green leafy vegetables are a natural source to recover the prevailing nutritional deficiency in developing countries (Arif et al., 2017). Common use of these vegetables in daily diet has been suggested as a cheapest source of micronutrients (Egbi et al., 2018). The WHO has recommended the consumption of at least 400 g of fruits and non-starchy vegetables, in order to meet the daily requirement of nutrients in human body (Arif et al., 2017).

Spinach is considered as the most desirable leafy vegetable in many countries around the globe, due to its richness in nutrients and a number of health-promoting compounds (Shi et al., 2016; Tang et al., 2019). Spinach leaves are rich with a number of vitamins, minerals, antioxidants, carotenoids, steroids, apocyanin, flavonoids, and *p*-coumaric acid (Avsar, 2011; Rashid et al., 2014). Its nitrates are helpful in maintaining blood pressure. Though its greater concentrations could also be toxic to human body, when nitrates are converted into nitrites (Mehri et al., 2021). It is also rich in vitamin K1, essential for blood clotting (Avsar, 2011; Rashid et al., 2014; Gunnars, 2019). Verma (2018) reported its potential uses in the remedy of urinary calculi and bowel and lung inflammation. It strongly prevents cancer cell proliferation, especially in breast cancers because of its high lutein and carotenoid contents (Avsar, 2011). The pharmacological properties of this leafy vegetable are due to its richness in a variety of phytochemicals, including its specific nutrients.

Crop variety and nutritional content of vegetable crops are particularly important for enhancing food and nutrition security on a global scale (Fresco and Baudoin, 2004). Moreover, it could be an important information for plant breeders to select improved varieties (Grant et al., 2008). However, the concentration of these nutritional components could be directly influenced by a wide range of climatic conditions (Natesh et al., 2017). The advent of molecular marker technology has transformed the study on crop’s quality (Lau et al., 2015). DNA-based markers have now been considered as a modern and more accurate tool to assess the genetic diversity irrespective to abrupt climatic influences. Among them, the functional markers (FMs) are directly linked to the allele of the trait of interest, gradually reducing the need of phenotypic validation (Varshney et al., 2005). Leafy vegetables, especially spinach, are rich in oxalates and nitrates. These important constituents have been found linked with the availability of essential nutrients in the crop (Joshi et al., 2021). Therefore, this study was conducted to assess the availability of mineral nutrients in genetically diverse spinach accessions using biochemical and functional marker strategies.

## Methodology

Seeds of 30 spinach accessions (Table 1) were obtained from the gene banks of the National Agricultural Research Center (NARC), Islamabad, Pakistan and the United States Department of Agriculture (USDA) and grown in the field area of Vegetable Research Institute, Ayub Agricultural Research Institute, Faisalabad, Pakistan. After the growth of 2 months, the leaves were collected, rinsed, dried, and grounded into fine powder and subjected to nutritional analysis from Food Technology Section, Post-Harvest Research Center, Ayub Agriculture Research Institute, Faisalabad, Pakistan.

**TABLE 1:** Geographical location of thirty spinach accessions for nutritional and genetic analyses.

S. No.	Accession	Country	Geographical location
1	163,310	Pakistan	30.4° N, 69.3° E
2	18,649	Pakistan	
3	18,650	Pakistan	
4	18,654	Pakistan	
5	18,655	Pakistan	
6	30,518	Pakistan	
7	30,526	Pakistan	
8	32,122	Pakistan	
9	32,618	Pakistan	
10	30,802	Pakistan	
11	19,339	Pakistan	
12	419,218	Hong Kong	22.3° N, 114.2° E
13	508,504	South Korea	35.9° N, 127.8° E
14	227,230	Japan	36.2° N, 138.3° E
15	647,853	Georgia	32.2° N, 82.9° W
16	647,858	Georgia	
17	163,309	India	20.59° N, 78.9° E
18	379,549	Macedonia	41.6° N, 21.7° E
19	212,119	Afghanistan	33.9° N, 67.7° E
20	321,020	Taiwan	23.7° N, 120.9° E
21	604,785	Mongolia	46.9° N, 103.8° E
22	266,926	Germany	51.2° N, 10.5° E
23	261,789	France	46.2° N, 2.2° E
24	181,964	Syria	34.8° N, 38.9° E
25	179,593	Belgium	50.5° N, 4.5° E
26	227,383	Iran	32.4° N, 53.7° E
27	648,942	China	35.9° N, 104.2° E
28	648,944	China	
29	184,379	United States	37.1° N, 95.7° W
30	171,860	Turkey	38.9° N, 35.2° E

### Proximate Analysis

Proximate analysis was performed to determine the moisture, ash, protein, fat, fiber, and carbohydrate and calorie contents of spinach accessions. Proximate contents were determined by following the methods of the Association of Official Analytical Chemists AOAC (2000).

*Moisture content:* Plant samples were weighed and dried in an oven at 105°C for 3 h. After drying, the sample was reweighed. The weights of sample before and after drying were used to calculate the moisture content of each sample (AOAC, 2000).

$$Moisture\left(\%\right)=\frac{W\,1-W\,2}{W\,1}\times 100$$

W1 = Weight of sample before drying.

W2 = Weight of sample after drying.

*Ash content:* About 5 g of plant sample was heated over low flame, till the fumes stop coming out. Then, it was placed in furnace overnight at 550°C and weighed once the ash turns to gray (AOAC, 2000).

$$Ash\left(\%\right)=\frac{W\,e\,i\,g\,h\,t\,o\,f\,A\,s\,h}{W\,e\,i\,g\,h\,t\,o\,f\,S\,a\,m\,p\,l\,e}\times 100$$

*Protein content:* It was determined by Kjeldahl’s method, including three main steps, i.e., digestion, distillation, and titration. About 0.5–1.0 g sample was put in a digestion flask. 5 g Kjeldahl’s catalyst and 200 ml concentrated H_2_SO_4_ were added in it. Then, another tube was prepared containing all the above chemicals, except sample. The flasks were placed in inclined position and heated gently until solution clears. After cooling, 60 ml of distilled water was added in it and the flask was immediately connected to digestion bulb on condenser with tip of condenser immersed in standard acid and 5–7 drops of mix indicator in receiver. The contents were mixed thoroughly and heated till all the ammonia was distilled. The receiver solution was removed and excess standard acid distilled was titrated with NaOH solution (AOAC, 2000).

$$Protein\left(\%\right)=\frac{\left(A-B\right)\times N\times 1.4007\times 6.25}{W}$$

A = Volume of 0.2 N HCl used sample titration (ml).

B = Volume of 0.2 N HCl used blank titration.

N = Normality of HCl.

W is the weight of sample (g).

1.4007 is the atomic weight of nitrogen.

6.25 is the protein-nitrogen conversion factor.

*Fat content:* About 3–5 g sample was weighed and wrapped in filter paper. The sample was extracted with petroleum ether into soxhlet extractor. After drying of solvent, the sample was reweighed to determine fat contents (AOAC, 2000).

$$Fat\left(\%\right)=\frac{W\,e\,i\,g\,h\,t\,o\,f\,F\,a\,t}{W\,e\,i\,g\,h\,t\,o\,f\,S\,a\,m\,p\,l\,e}\times 100$$

*Fiber content:* A total of 5 g of plant material was mixed with 200 ml of 1.25% sulfuric acid, heated for 30 min and filtered. The residue was washed with distilled water till it becomes acid free. Then, boiled in 200 ml of 1.25% of sodium hydroxide for 30 min, filtered, and washed with distilled water to make it alkaline free. The residue was rinsed once with 10% hydrochloric acid, twice with ethanol, and thrice with petroleum ether. Then, the residue was dried in oven at 105°C. After cooling, it was ignited in a muffle furnace for 90 min at 550°C to get the weight of fiber (AOAC, 2000).

*Carbohydrate content:* (Pearson, 1976; James, 1995; Otitoju, 2009).

Carbohydrates(%)=100-(M⁢o⁢i⁢s⁢t⁢u⁢r⁢e+A⁢s⁢h+P⁢r⁢o⁢t⁢e⁢i⁢n+F⁢a⁢t+F⁢i⁢b⁢e⁢r)

*Energy content:* Energy contents were determined using bomb calorimeter. Small pellets of sample were produced on a briquette press followed by bombing with 25 atmospheric pressure (Anon, 1970).

### Mineral Analysis

Mineral contents were determined by wet acid digestion method followed by atomic absorption spectroscopy (AOAC, 1990). About 2 g of powdered plant material was taken in crucible and ignited for 6 h in a muffle furnace at 550°C. The ignited residue was dissolved in 10% nitric acid (10 ml) and heated slowly for 20 min. Then, the filtrate of this material was used for the determination of mineral contents by atomic absorption spectrophotometry. The technique was used for the quantification of both the macronutrients (sodium, calcium, and potassium) and micronutrients (zinc, iron, and manganese) of spinach accessions.

### Oxalate Content Determination

A total of 1 g dried plant sample was boiled for 25 min in 150 ml of water containing 27.5 ml, 6 M HCl along with 2 drops of octanol. After cooling, the mixture was diluted up to 250 ml and filtered. The filtrate of 10 ml was evaporated at 40–45°C and redissolved in 0.01 M 10 ml sulfuric acid. A solution of 0.5 mM K_2_Cr_2_O_7_ and 0.25 mM MnCl_2_ was prepared as blank solution. A total of 10 ml solution (1 ml extracted sample + 9 ml blank solution) was taken for each sample, incubated for 60 min, and then analyzed using UV-Vis spectrophotometer for the determination of oxalate contents in the sample (Popoola et al., 2014).

### Nitrate Content Determination

A total of 20 g of each sample was homogenized with 100 ml of deionized water and centrifuged at 3,000 rpm for 10 min. Supernatant was collected for spectrophotometric analysis. The sample was diluted to 1/10th and 1 ml solution was added to an ion-exchange solid phase extraction (SPE) cartridge, which was preconditioned with 2 ml of deionized water. The solution was then washed out with 10 ml of deionized water, followed by 0.5 M NaCl solution to elute the nitrate components. 5 ml elute was collected for spectrometry analysis. Nitrate in aqueous solution results in an absorbance peak at wavelength of 220 nm and no absorbance at 275 nm. The net absorbance (Abs) for nitrate was calculated using the following equation (Yu et al., 2018) and converted into the nitrate concentration *via* a calibration curve, set by a series of standard solutions with concentrations from 0 to 25 μg/ml. The standard solutions, including a blank, were also prepared in 0.5 M sodium chloride solution.

$$N\,e\,t\,A\,b\,s\,f\,o\,r\,N\,O_{3}^{-}=\left(A\,b\,s_{220\,n\,m}-2\right)\times A\,b\,s_{275\,n\,m}$$

The derived concentration was multiplied by the total weight of homogenized sample (120 g) and dilution factors and divided by the weight of plant sample (20 g) to become the total nitrate content (μg/g).

### Development of Functional Markers for Genetic Analysis

A total of 110 primers were designed related to specific enzymes involved in oxalate metabolism and downy mildew disease resistance gene (fungal disease common in spinach) (Correll et al., 1994). Already reported sequences of genes in the National Center for Biotechnology Information (NCBI) database were used to synthesize primers for amplification of genes. Sequences of functionally characterized genes for ten important enzymes, involved in oxalate metabolism (i.e., glycolate oxidase, malate dehydrogenase, ascorbate peroxidase, isocitrate lyase, malate synthase, citrate synthase, ascorbate oxidase, oxalyl-CoA synthetase, formate dehydrogenase, and oxalate-CoA ligase), were used to develop 100 primer pairs to assess the genetic variability of spinach accessions. In addition, the sequence of gene responsible for regulation of downy mildew resistance in spinach (i.e., glucose-6-phosphate dehydrogenase) was also used to develop ten primer pairs for genetic characterization of accessions (Supplementary Table 1).

### Deoxyribonucleic Acid Extraction

Seedlings of spinach were used to extract DNA by cetyltrimethylammonium bromide (CTAB) method (Doyle, 1990). 1 g of the plant material was put in pestle and mortar and crushed in 600 μl CTAB buffer. After fine grinding, the mixture was transferred to 1.5 μl Eppendorf tube, homogenized, and placed in water bath at 60°C for 30 min. The mixture was cooled down at room temperature for 5–7 min. After normalizing temperature, 600 μl isoamyl alcohol chloroform solution was added in the mixture and centrifuged at 13,000 rpm for 10 min. The supernatant was carefully transferred to another tube for further processing. 300 μl ice cold isopropanol was added in the tube containing supernatant solution and the mixture was kept at −20°C for 30 min. After thawing, the mixture was centrifuged at 13,000 rpm for 10 min for the formation of DNA pellet at the bottom of tube. After pellet formation, the aqueous solution was carefully removed and the pellet was washed with 200 μl of 70% ethanol solution by centrifuging at 13,000 rpm for 10 min. After washing of DNA pellet, the solution was carefully removed from the tube and the pellet was allowed to dry for about 2 h, till the smell of ethanol was completely eliminated from the tube. After complete drying, the pellet was dissolved in 100 μl of TE buffer and vortex for 1 min. After complete dissolving, 1 μl RNase solution was added in the DNA extract and vortex for 1 min to degrade all the RNA from it. DNA quality and quantity were determined by gel electrophoresis and NanoDrop spectrophotometer technique, respectively (Abdel-Latif and Osman, 2017). The DNA extract was stored at −20°C till further use.

### Polymerase Chain Reaction Amplification

Reaction mixture for PCR was prepared with the volume of 20 μl by making a solution of PCR master mix, nuclease-free water, DNA sample, and forward and reverse primers. The mixture was prepared on ice. Amplification of desired DNA template was performed in PCR thermal cycler (Bio-Rad, T100 Thermal Cycler).

### Gel Electrophoresis

Agarose gel of 1% concentration was prepared to run the DNA samples. Solidified gel within its casting tray was placed in gel buffer tank (Wealtec Corporation, Model: mini GES), filled with TAE buffer (Cole and Tellez, 2002). Each DNA sample (5 μl) along with negative control was loaded in the respective wells of the gel. DNA ladder of 100 base pairs (bps), with 13 discrete DNA fragments ranging from 100 to 3,000 bp, was used as the standard to determine the DNA fragment size on gel electrophoresis. After running samples, the gel was taken out from the buffer tank and observed under UV light (UV transilluminator, Cleaver Scientific Ltd.) to visualize the variation among DNA bands.

### Statistical Analysis

The nutritional and genetic variabilities of spinach accessions were assessed by statistical software RStudio version 1.3.1093. The ANOVA was performed for nutritional data with 0.1, 1, and 5% levels of significance. The broad sense heritability (h^2^b) was calculated as σ^2^_g_/σ^2^_p_, representing low and high values as < 50 and > 80%, respectively (Riaz et al., 2021). The genetic advance was calculated as, GA = h^2^b × σ_p_ × K, where σ_p_ and K represented phenotypic SD and standardized selection differential constant, respectively, at 5% selection intensity (Riaz et al., 2021). The genotypic coefficient of variation (GCV) and phenotypic coefficient of variation (PCV) were calculated by the method of Ogunniyan and Olakojo (2014). Genotypic and phenotypic correlations were calculated for all the nutritional characters and represented in the form of heat map diagram. Path coefficient analysis was performed to evaluate the direct and indirect effects of the most highly correlated nutrients on oxalate contents (Riaz et al., 2021). Functional marker data of spinach accessions, based on DNA banding pattern, was analyzed through correlation coefficient to determine the genetic relationship among accessions. The genetic diversity of accessions was determined by cluster analysis of functional marker data and represented in the form of heat map diagram (Cui et al., 2020).

## Results and Discussion

### Proximate Analysis

Proximate composition is essential to assess the nutritional significance of plants, especially leafy vegetables (Oyugi, 2016). Significant differences were observed among the nutritional contents of accessions. Most of the variation was observed among moisture and carbohydrate contents of accessions compared to rest of the contents. Moisture content of accessions ranged from 82 in accession 163,309 (from India) to 84.69% in accession 30,526 (from Pakistan). This high level of moisture in leafy vegetables could be helpful for the activity of a number of water-soluble enzymes required for good metabolism of plants (Iheanacho and Ubebani, 2009). In these results, above 80% moisture content was observed in all of the accessions, showing its active metabolism due to aqueous environment. Similar value of moisture was reported by Hussain et al. (2009) in spinach compared to other vegetables studied. The previous findings also revealed that the abovementioned accessions from Pakistan and India, in this study, were found to be good in terms of moisture contents and involvement in active metabolism.

Fibers are important dietary constituents in terms of good impact on digestion and waste elimination. Also, these can help in prevention of a number of digestive and cardiovascular disorders (Sodipo et al., 2000). Among all the studied spinach accessions, fiber contents ranged from 2.10 in accession 648,942 (China) to 2.34% in accession 30,802 (Pakistan). The results showed the importance of Asian spinach accessions due to its richness in fiber contents. The reason might be the better adaptability of these accessions in the local environment. Compared to a previous report about *Cucurbita* spp. (Nwofia et al., 2012), all the accessions of spinach were found to be rich in fibers. Fat contents also varied significantly from 0.51 to 0.75% among all the spinach accessions. High quantity of fats can lead toward obesity or overweight, but its moderate amount in diet is a very good source of energy (Sodipo et al., 2000; Erukainure et al., 2011). Present results have indicated few accessions with intermediate, but slightly different levels of fats. These accessions included 227,230 (Japan), 321,020 (Taiwan), 227,383 (Iran), and 647,858 (Georgia). This slight variation might be due to different origins of spinach accessions compared to that of local origin.

Ash contents in a plant represent its richness in mineral elements (Oyugi, 2016). Significant variation was found among ash contents of spinach accessions. Maximum percentage of ash was observed in accessions of China (648,942), Pakistan (163,310, 30,802), and Germany (266,926). These values were slightly lower than that reported by Galla et al. (2017) in Indian spinach. There was also variation in protein and calorie contents within the spinach accessions. Protein contents varied from 2.41 to 2.64%, while caloric values were in the range of 11.84 to 16.76 MJ/kg. Higher level of protein and calories shows the superiority of a crop on others (Oyugi, 2016). According to Ene-Obong (1992), this diet is considered to be nutritionally fit, containing high protein and caloric values. In these results, accessions 163,310 (from Pakistan) and 648,942 (from China) were found to be rich in terms of both the protein and caloric values. Like fats and proteins, digestible carbohydrates are also an important source of energy (Oyugi, 2016). In contrast to other two energy sources (fats and proteins), more variation was observed in terms of carbohydrate content. Few accessions of Pakistan (18,654, 32,122, and 19,339), India (163,309), China (648,942), United States (184,379), and Turkey (171,860) were found to be rich in carbohydrates (i.e., above 10%). Variation in nutritional composition of plants might be due to the influence of different environmental conditions (Odhav et al., 2007).

### Mineral Analysis

Minerals are the important constituents of diet for good health and proper growth. Some of the minerals are integral parts of blood, teeth, muscles, and bones. Hence, their specific amount is required for proper functioning of body systems (Soetan et al., 2010). Though in trace quantities, micronutrients play a vital role in catalytic and balancing activity of plants (Jan et al., 2011). Leafy vegetables are considered as the richest source of minerals, especially iron, calcium, sodium, potassium, zinc, and manganese (Odhav et al., 2007). Significant variation was observed among mineral composition of spinach accessions. Among micronutrients, iron (Fe) content varied greatly, followed by manganese (Mn) and zinc (Zn) contents. Variation in Fe contents ranged from 27.1 (accession 184,379, United States) to 79.9 ppm (accession 181,964, Syria). Iron, being an important element of blood hemoglobin, requires for proper functioning of circulatory and central nervous system as well (Hussain et al., 2010). Accessions 32,618 (Pakistan) and 181,964 (Syria) were found to be richest in iron. Therefore, these accessions could be selected to fulfill iron requirement through leafy vegetables. Moreover, iron content of these accessions was found to be superior than that of previous reports about spinach (Kuti and Torres, 1996; Galla et al., 2017).

Zinc is required for better growth and proper functioning of immune and reproductive systems (Black, 2003; Hambidge, 2006). The quantity of zinc varied from 3 to 8 ppm among spinach accessions. Few accessions from Pakistan (163,310, 18,649, and 18,655) and one from India (163,309) were having high zinc contents. Highest value of Mn (8.2 ppm) was found in accession 163,310 (Pakistan), while lowest value of Mn (2.9 ppm) was found in accession 227,230 (Japan). The values of these micronutrients were even greater than that of previous reports about spinach and few other vegetables from Cucurbitaceae (Hussain et al., 2010), suggesting the use of these accessions for proper functioning of body.

Quantities of macronutrients also varied greatly among spinach accessions. Significant differences were found in terms of potassium (K) contents, followed by calcium (Ca) and sodium (Na) (Table 2). All these minerals have specific roles in human body. Calcium is an important dietary constituent as being helpful in osteogenesis (Hodges et al., 1978). Potassium is helpful in controlling the risk of stroke and hypertension (National Research Council [NRC], 1989). Highest value of potassium (4,000 ppm) was observed in accession 163,310 from Pakistan, while lowest value of potassium (3,240 ppm) was observed in accession 419,218 from Hong Kong. Calcium concentration varied from 550 in accessions 30,802 (Pakistan) and 227,230 (Japan) to 1,070 ppm in accession 163,310 (Pakistan). Few Pakistani accessions (163,310, 18,649, and 18,655) showed even greater Ca and K contents than that of a previous report about chaya and spinach (Kuti and Torres, 1996). Also, the same accessions showed higher contents of Na. Sodium content among accessions ranged from 420 to 876 ppm. In contrast to one previous report (Nwofia et al., 2012) about spinach of Nigeria, quantity of Na was comparatively lesser. The variation in nutritional contents might be due to the influence of environmental factors, harvest time, or farming practices, which ultimately leads toward the overall genetic diversity (Odhav et al., 2007).

**TABLE 2:** ANOVA for nutritional contents of spinach accessions.

	Moisture	Fat	Fiber	Protein	Ash	CHO	Calories	Na	Ca	K	Zn	Fe	Mn	Oxalates	Nitrates
Mean Sq	0.12***	0.02***	0.05***	0.03***	0.03***	3.06***	0.17***	5.8***	264.6***	792***	0.05***	0.23**	0.02**	0.13***	2,362.9
Mean	0.06	0.01	0.02	0.01	0.01	1.53	0.09	2.88	132.30	396.03	0.02	0.11	0.01	0.07	1,181.4
Standard Error	0.05	0.01	0.02	0.01	0.01	0.21	0.04	0.11	0.78	1.45	0.01	0.05	0.01	0.03	0.02
σ^2^_e_	0.01	0.00	0.00	0.00	0.00	0.13	0.01	0.04	1.82	6.31	0.00	0.01	0.00	0.00	0.02
σ^2^_g_	0.53	0.01	0.01	0.01	0.05	4.10	1.99	202.31	257.36	543.26	0.02	1.44	0.02	0.03	653.2
σ^2^_ph_	0.54	0.01	0.01	0.01	0.05	4.23	2.00	202.35	259.18	549.57	0.02	1.45	0.02	0.03	657.7
ECV	0.11	1.43	1.24	0.75	0.71	4.06	0.53	0.31	1.62	0.70	4.16	1.67	3.33	0.34	0.54
GCV	0.88	11.13	3.41	3.07	9.15	22.98	9.89	22.48	19.24	6.45	25.44	22.38	26.70	1.28	30.7
PCV	0.89	11.24	3.64	3.17	9.18	23.34	9.91	22.48	19.31	6.49	25.79	22.44	26.90	1.32	30.6
H^2^B	0.99	0.98	0.88	0.94	0.99	0.97	1.00	1.00	0.99	0.99	0.97	0.99	0.99	0.93	0.99
Genetic Advance	1.50	0.14	0.15	0.15	0.44	4.11	2.90	29.30	32.93	47.74	0.27	2.46	0.29	0.33	12.4
Genetic Advance % Mean	1.80	22.70	6.58	6.13	18.79	46.62	20.35	46.30	39.50	13.22	51.69	45.98	54.60	2.54	25.5

CHO, Carbohydrates; Na, Sodium; Ca, Calcium; K, Potassium; Zn, Zinc; Fe, Iron; Mn, Manganese; Mean Sq, mean squares; σ^2^_ph_, phenotypic variance; σ^2^_g_, genotypic variance; σ^2^_e_, environmental variance; PCV, phenotypic coefficient of variation; GCV, genotypic coefficient of variation; ECV, environmental coefficient of variation; H^2^B (%), broad sense heritability. ** Significant, *** Highly Significant.

### Oxalate and Nitrate Contents

Leafy vegetables are rich in oxalates and nitrates. However, its higher concentration greatly affects the mineral absorption. In this study, maximum oxalate contents (13.16 g/100 g dw) were found in accession 30,526 from Pakistan. The mineral composition of this accession was comparatively lower than other accessions, while another accession from Pakistan (163,310) was found to be lesser in terms of oxalate contents, but rich in minerals (Na = 876 ppm, Ca = 1,070 ppm, K = 4,000 ppm, Zn = 80 ppm, Fe = 51 ppm, and Mn = 8.2 ppm) and nitrates (2,549 ppm) as well. This also represented the effect of varied oxalate concentration on mineral and nitrate contents. Nitrates and oxalates have been considered as the antinutritional phytochemicals. However, the crops with good oxalate metabolism and better nitrogen use efficiency can reduce the harmful impacts of these antinutritional phytochemicals (Uusiku et al., 2010). In this study, the accessions with greater macro- and micronutrients represented lesser oxalates, showing its better metabolism, which were also assessed through genetic marker study. Moreover, the accessions should have better nitrogen use efficiency that represented with positive relationship of nitrates with other nutritional factors. These findings were also supported by the facts reported by Natesh et al. (2017).

### Genetic Variability

The nutritional data was evaluated for variance analysis and the values of mean, variance, coefficient of variation, heritability, and genetic advance are given in Table 2 to represent the significance of various nutritional parameters. Significant variation was observed among nutritional contents of different spinach accessions. The ANOVA showed nearly equal phenotypic and genotypic variances compared to environmental variance, which was very low for all the nutritional traits. Similar observations were reported by Riaz et al. (2021) showing comparatively lesser influence of environment on the nutritional values. The phenotypic and genotypic coefficients of variations were higher for nitrates, manganese, zinc, iron, sodium, and carbohydrates. Low phenotypic and genotypic coefficients of variation were observed for moisture and oxalates. However, the environmental coefficient of variation was lower for most of the nutritional traits. Genetic heritability was higher for calories and sodium.

### Correlation Analysis

**FIGURE 1:**
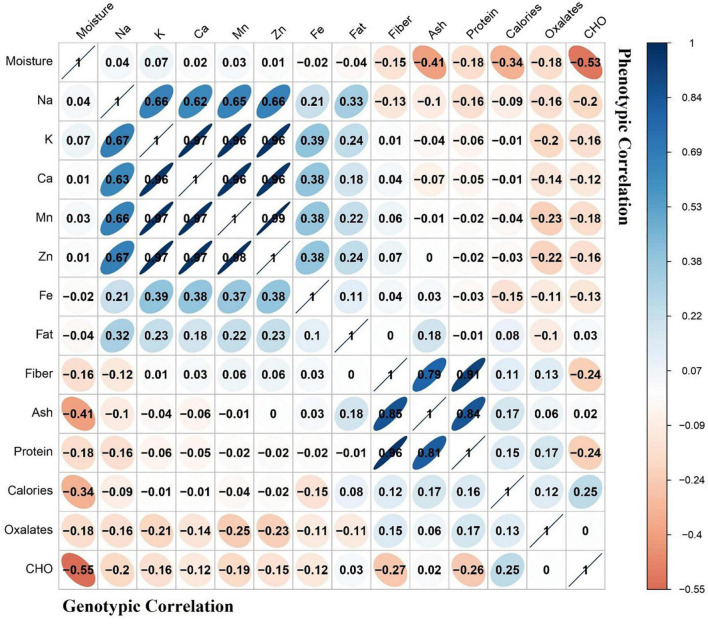
Heat map indicating phenotypic and genotypic correlations of nutritional contents. Dark blue color is the representation of greater correlation level.

Correlation analysis is a technique that is helpful in selection of positive correlated traits by giving positive or negative impacts on its correlated traits (Manggoel et al., 2012). Moreover, this analysis is helpful when highly heritable traits are linked with important nutritional contents of the leafy crops. A heat map of genotypic and phenotypic correlation coefficients was constructed among various nutritional parameters (Figure 1). The genotypic correlation coefficients were very closely correlated to the phenotypic correlation coefficients. Maximum positive correlation (97%) was observed among calcium, manganese, and zinc minerals, while negative correlation (50%) was found between moisture and carbohydrates.

### Genotypic Pathway Analysis

**FIGURE 2:**
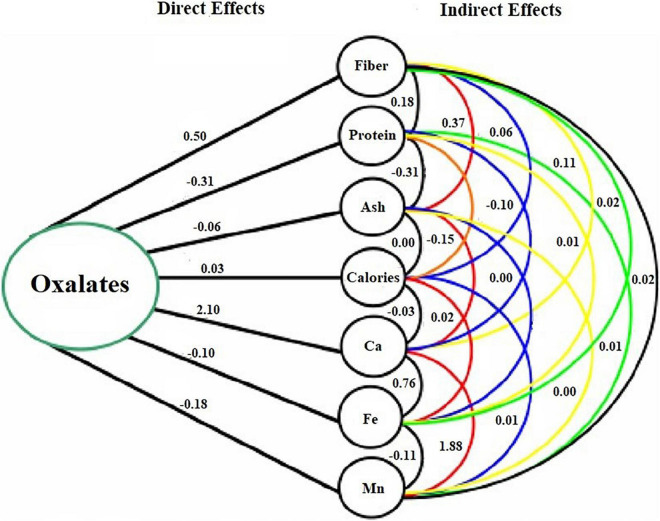
Path diagram representing direct and indirect effects of nutrients on oxalates.

Besides the importance of correlation coefficients in determining the linkage of traits, the information could be a little misleading, if the observed higher correlation coefficient is due to the indirect effects of traits (Manggoel et al., 2012). Therefore, a path coefficient analysis, in addition, was performed to determine the direct and indirect effects of interrelated nutritional traits (Table 3). Among all the observed nutritional traits, seven highly linked traits with greater values of direct effects on oxalates were considered to build a path diagram, representing its relationship of direct and indirect effects with that of oxalate contents (Figure 2). The oxalate concentration in spinach accessions was found to be greatly linked with calcium concentration, as these two constituents are usually found as calcium oxalates in leafy vegetables (Ferreira et al., 2018). The least effect on oxalates was shown by proteins of spinach accessions. On the other hand, calcium and manganese had indirect effects on oxalate contents of spinach.

**TABLE 3:** Pathway analysis showing direct and indirect effects of nutritional content on oxalates of spinach accessions.

Variables	Indirect effects to oxalates													Total effects		Total correlation
	1	2	3	4	5	6	7	8	9	10	11	12	13	Direct	Indirect	
Moisture		0.00	–0.08	0.06	0.02	0.15	–0.01	0.00	0.04	0.01	–0.05	0.00	–0.01	–0.33	0.14	–0.19
Fat	0.02		0.00	0.01	–0.01	–0.01	0.00	0.04	0.38	0.04	–0.59	–0.01	–0.04	0.07	–0.18	–0.11
Fiber	0.05	0.00		–0.30	–0.05	0.07	0.00	–0.01	0.09	0.00	–0.17	0.00	–0.01	0.50	–0.34	0.16
Protein	0.06	0.00	0.48		–0.05	0.07	0.01	–0.02	–0.12	–0.01	0.06	0.00	0.00	–0.31	0.49	0.18
Ash	0.14	0.01	0.42	–0.26		–0.01	0.01	–0.01	–0.15	–0.01	–0.01	0.00	0.00	–0.06	0.12	0.06
CHO	0.18	0.00	–0.14	0.08	0.00		0.01	–0.02	–0.26	–0.03	0.40	0.01	0.03	–0.27	0.27	0.00
Calories	0.11	0.01	0.06	–0.05	–0.01	–0.07		–0.01	–0.03	0.00	0.07	0.02	0.01	0.03	0.10	0.13
Na	–0.01	0.02	–0.06	0.05	0.01	0.05	0.00		1.33	0.11	–1.63	–0.02	–0.12	0.11	–0.28	–0.17
Ca	–0.01	0.01	0.02	0.02	0.00	0.03	0.00	0.07		0.16	–2.35	–0.04	–0.17	2.10	–2.25	–0.15
K	–0.03	0.02	0.01	0.02	0.00	0.04	0.00	0.07	2.05		–2.36	–0.04	–0.18	0.17	–0.38	–0.21
Zn	–0.01	0.02	0.04	0.01	0.00	0.04	0.00	0.07	2.06	0.17		–0.04	–0.18	–2.40	2.17	–0.23
Fe	0.01	0.01	0.02	0.01	0.00	0.03	0.00	0.02	0.80	0.07	–0.92		–0.07	–0.10	–0.02	0.12
Mn	–0.01	0.02	0.03	0.01	0.00	0.05	0.00	0.07	2.05	0.16	–2.42	–0.04		–0.18	–0.07	0.25

*CHO, Carbohydrates; Na, Sodium; Ca, Calcium; K, Potassium; Zn, Zinc; Fe, Iron; Mn, Manganese.*

### Functional Marker Analysis

Oxalates are among the active metabolic components of leafy vegetables, especially spinach. It readily affects the absorption of mineral ions in plant tissue. The active metabolic cycle of oxalates is regulated by a number of metabolic enzymes, which maintain its concentration in plant tissue (Horner et al., 2005). Therefore, this study included the use of functional molecular markers related to the genes involved in oxalate metabolism in order to relate the oxalate contents and mineral availability in spinach accessions.

Leaves of spinach are rich in minerals, especially iron, calcium, potassium, and sodium. Potassium, sodium, and calcium minerals are usually found in association with oxalates either as soluble salts or as insoluble crystals of calcium oxalate (Ferreira et al., 2018). Oxalates are largely distributed in spinach, accounting for about 5–15% of dry weight of spinach leaves. A number of functional roles have been reported for oxalates in calcium regulation, ion balancing (Na, K), and detoxification of heavy metals, especially in case of leafy vegetables (Botstein et al., 1980; Amom and Nongdam, 2017; Dhutmal et al., 2018). Apart from positive roles, excessive accumulation of oxalates in plants could lead toward negative impacts on human health (Cai et al., 2018). Therefore, a balance should be maintained between anabolic and catabolic pathways of oxalate. A number of enzymes are involved in anabolic and catabolic pathways of oxalate. The presence or absence of its genes could be considered a criterion for selection of improved varieties. Another important consideration for breeders is the development of disease-resistant varieties. Significant variation was observed among genetic profile of accessions, especially with oxalate-related genetic markers.

### Allele Number and Size

**FIGURE 3:**
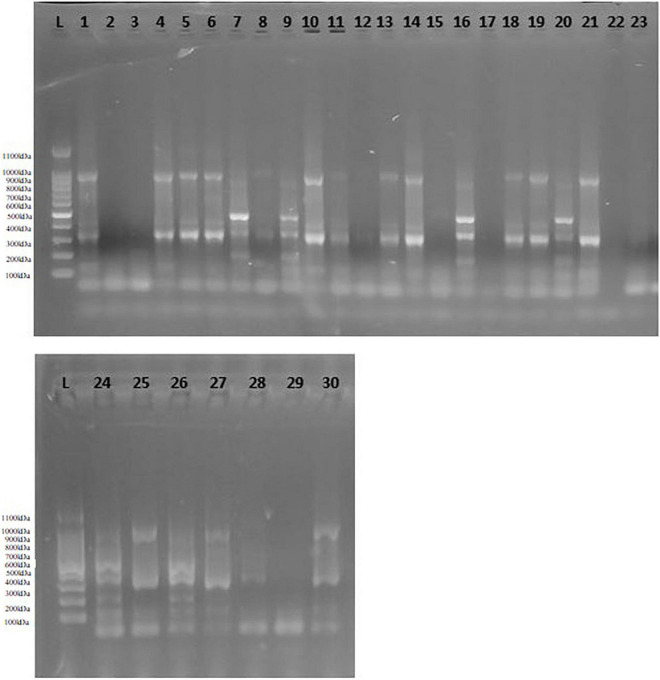
Allelic profile of oxalate-CoA ligase among spinach accessions generated by primer pair OxLig-1. L = 100 bp ladder, 1 = 321,021, 2 = 19,339, 3 = 30802, 4 = 18,649, 5 = 18,654, 6 = 30,518, 7 = 32,618, 8 = 227,230, 9 = 379,549, 10 = 18,650, 11 = 32,122, 12 = 508,504, 13 = 647,858, 14 = 604,785, 15 = 163,309, 16 = 163,310, 17 = 419218, 18 = 212,119, 19 = 647853, 20 = 30,526, 21 = 18,655, 22 = 266,926, 23 = 261,789, 24 = 181,964, 25 = 179,593, 26 = 227,383, 27 = 648,942, 28 = 648,944, 29 = 184,379, 30 = 171,860.

Spinach accessions showed a great deal of genetic variation based on functional molecular markers. Figure 3 displays the variation in banding pattern of oxalate-CoA ligase gene among spinach accessions. The allelic variation was represented in the form of differences in allele number and sizes (Supplementary Table 1). Number of alleles ranged from 2 to 21. Maximum number of alleles (21) was represented by the DNA product form from primer pair OxySyn-1, followed by AscPerox with 20 allele number. Minimum number of alleles was formed as a result of amplification by primer pairs GlyOx-7 and GlyOx-9. Size of amplified product ranged from 73 to 994 bp. Maximum product size (994 bp) was formed as a result of amplification by primer set AscOx-3, while minimum product length (73 bp) was shown by primer set G6PDH-2.

In these results, genetic variation was assessed in terms of DNA banding pattern. Out of 130 bands, 115 bands were found to be polymorphic representing 89% polymorphism. Most of the primers related to oxalate biosynthesis genes were found to be highly polymorphic. Functional marker AscPerox-1 was found to be highly polymorphic among all with 0.92 polymorphic information content (PIC) value and heterozygosity. It was followed by IsoLy-1 with 0.91 PIC value and heterozygosity. Minimum value of PIC (0.32) and heterozygosity (0.26) was represented by primer pair AscOx-2 (Supplementary Table 1). In a similar study, Avsar (2011) used twenty-five sequence-related amplified polymorphism (SRAP) markers for genetic characterization of ninety-five Turkish spinach accessions. The banding pattern showed 123 amplified bands, out of which 67 bands were polymorphic giving rise about 54% genetic polymorphism. In another study, Goktay (2015) characterized 176 spinach accessions through 15 simple sequence repeat (SSR) markers with 98% polymorphism. The banding pattern showed 57 polymorphic bands out of 58 bands. Hu et al. (2007) reported genetic variability of 48 spinach germplasm based on 59 target region amplification polymorphism (TRAP) markers. However, the obtained polymorphism was low (19.5%) compared to other reports. The comparison revealed the importance of functional markers as more polymorphic. Similar opinion was stated by Salgotra and Stewart (2020) about importance of functional markers due to its specificity to a particular trait.

### Correlation and Cluster-Based Relationship

Table 4 represents the correlation of 30 spinach accessions based on observed genetic polymorphism. Accessions 508,504 and 184,379 were found to be highly correlated representing 91% similarity. Minimum positive correlation (1%) was shown by accessions 18,649 (with 32,618 and 181,964), 18,650 (with 379,549 and 648,942), and 163,309 (with 32,618 and 181,964). Few accessions were found to be negatively correlated in terms of genetic polymorphism. Maximum negative correlation (17%) was represented by accessions 266,926 (with 212,119 and 6,489,440) and 32,618 (with 181,964, 18,649, 18,654, and 30,526), while minimum correlation of about 1% was found in accessions 227,383, 508,504, 227,383, and 19,339 with reference to each other. Also, similar negative correlation was shown by accession 18,649 with 32,618 and 181,964.

**TABLE 4:** Correlation of thirty spinach accessions based on functional marker analysis.

	1	2	3	4	5	6	7	8	9	10	11	12	13	14	15	16	17	18	19	20	21	22	23	24	25	26	27	28	29	30
1	1																													
2	0.07	1																												
3	0.20	0.01	1																											
4	0.17	0.01	0.54	1																										
5	–0.02	0.13	0.12	0.07	1																									
6	0.37	0.02	0.24	0.41	0.16	1																								
7	0.17	–0.07	0.62	0.66	0.13	0.33	1																							
8	0.36	0.22	0.07	0.12	0.43	0.24	0.12	1																						
9	0.07	–0.01	0.04	0.22	0.28	0.21	0.05	0.32	1																					
10	–0.02	–0.08	0.41	0.40	0.20	0.25	0.40	–0.03	0.18	1																				
11	0.11	0.32	–0.08	–0.06	0.17	0.00	–0.03	0.35	0.08	–0.02	1																			
12	0.31	–0.06	–0.02	0.07	0.09	0.23	–0.02	0.17	0.36	0.08	0.10	1																		
13	0.19	0.03	0.10	0.02	–0.06	0.12	0.03	0.19	–0.06	–0.12	–0.01	0.23	1																	
14	–0.07	0.11	0.05	–0.02	0.11	–0.14	–0.12	0.09	–0.03	–0.06	0.23	0.07	0.09	1																
15	0.28	0.11	0.18	0.11	–0.05	0.20	0.11	0.28	–0.05	–0.09	0.04	0.20	0.77	0.09	1															
16	0.15	–0.02	0.13	0.12	0.12	0.16	0.12	0.21	0.18	0.02	0.04	0.39	0.26	0.22	0.23	1														
17	0.08	–0.08	0.27	0.27	0.14	0.17	0.31	0.13	0.01	0.35	–0.01	0.08	0.29	0.14	0.22	0.37	1													
18	0.15	0.12	0.01	0.03	0.35	0.10	0.04	0.41	0.19	–0.05	0.32	0.05	–0.10	0.28	–0.07	0.09	–0.03	1												
19	–0.08	–0.17	–0.12	–0.17	–0.07	–0.11	–0.17	–0.08	–0.08	–0.14	–0.13	–0.02	–0.05	0.31	–0.04	–0.10	0.35	–0.11	1											
20	0.16	0.00	0.07	0.00	–0.07	0.10	0.00	0.16	–0.07	–0.13	–0.03	0.29	0.91	0.06	0.70	0.22	0.24	–0.10	–0.05	1										
21	0.11	0.02	0.24	0.30	0.17	0.22	0.23	0.17	0.03	0.30	0.03	0.17	0.32	0.07	0.24	0.42	0.73	0.05	–0.07	0.35	1									
22	0.17	–0.07	0.62	0.66	0.13	0.33	0.75	0.12	0.05	0.40	–0.03	–0.02	0.03	–0.12	0.11	0.12	0.31	0.04	–0.17	0.00	0.23	1								
23	0.36	0.22	0.07	0.12	0.43	0.24	0.12	0.75	0.32	–0.03	0.35	0.17	0.19	0.09	0.28	0.21	0.13	0.41	–0.08	0.16	0.17	0.12	1							
24	0.07	–0.01	0.04	0.22	0.28	0.21	0.05	0.32	0.75	0.18	0.08	0.36	–0.06	–0.03	–0.05	0.18	0.01	0.19	–0.08	–0.07	0.03	0.05	0.32	1						
25	–0.02	–0.08	0.41	0.40	0.20	0.25	0.40	–0.03	0.18	0.75	–0.02	0.08	–0.12	–0.06	–0.09	0.02	0.35	–0.05	–0.14	–0.13	0.30	0.40	–0.03	0.18	1					
26	0.11	0.32	–0.08	–0.06	0.17	0.00	–0.03	0.35	0.08	–0.02	0.75	0.10	–0.01	0.23	0.04	0.04	–0.01	0.32	–0.13	–0.03	0.03	–0.03	0.35	0.08	–0.02	1				
27	0.15	0.12	0.01	0.03	0.35	0.10	0.04	0.41	0.19	–0.05	0.32	0.05	–0.10	0.28	–0.07	0.09	–0.03	0.75	–0.11	–0.10	0.05	0.04	0.41	0.19	–0.05	0.32	1			
28	–0.08	–0.17	–0.12	–0.17	–0.07	–0.11	–0.17	–0.08	–0.08	–0.14	–0.13	–0.02	–0.05	0.31	–0.04	–0.10	0.35	–0.11	0.75	–0.05	–0.07	–0.17	–0.08	–0.08	–0.14	–0.13	–0.11	1		
29	0.16	0.00	0.07	0.00	–0.07	0.10	0.00	0.16	–0.07	–0.13	–0.03	0.29	0.91	0.06	0.70	0.22	0.24	–0.10	–0.05	0.75	0.35	0.00	0.16	–0.07	–0.13	–0.03	–0.10	–0.05	1	
30	0.11	0.02	0.24	0.30	0.17	0.22	0.23	0.17	0.03	0.30	0.03	0.17	0.32	0.07	0.24	0.42	0.73	0.05	–0.07	0.35	0.75	0.23	0.17	0.03	0.30	0.03	0.05	–0.07	0.35	1

*Key: 1 = 163,310; 2 = 18,649; 3 = 18,650; 4 = 18,654; 5 = 18,655; 6 = 30,518; 7 = 30,526; 8 = 32,122; 9 = 32,618; 10 = 30,802; 11 = 19,339; 12 = 419,218; 13 = 508,504; 14 = 227,230; 15 = 647,853; 16 = 647,858; 17 = 163,309; 18 = 379,549; 19 = 212,119; 20 = 321,020; 21 = 604,785; 22 = 266,926; 23 = 261,789; 24 = 181,964; 25 = 179,593; 26 = 227,383; 27 = 648,942; 28 = 648,944; 29 = 184,379; 30 = 171,860. Highlighted values represent maximum and minimum correlation coefficient among accessions.*

**FIGURE 4:**
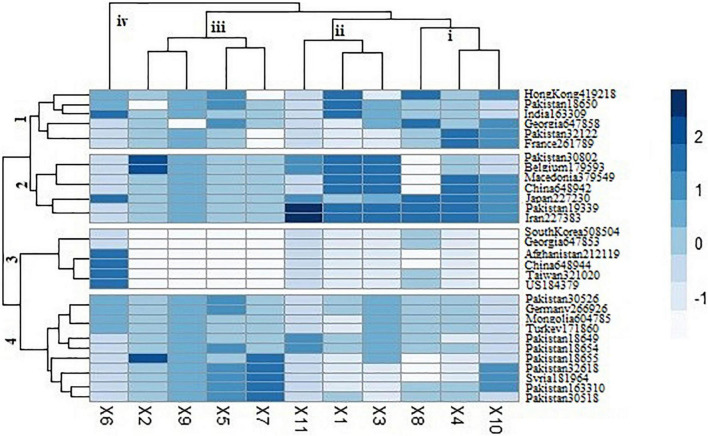
Heat map clustering of spinach accessions based on functionally characterized genes. X1 = Glycolate oxidase, X2 = Oxalyl CoA Synthetase, X3 = Glucose-6-Phosphate Dehydrogenase, X4 = Malate Dehydrogenase, X5 = Ascorbate Peroxidase, X6 = Iso-citrate lyase, X7 = Formate Dehydrogenase, X8 = Oxalate CoA Ligase, X9 = Malate Synthase, X10 = Citrate Synthase, X11 = Ascorbate Oxidase. Dark blue color is the representation of greater heterozygosity level.

Cluster analysis was performed among spinach accessions based on genetic profile and represented in the form of heat map (Figure 4). This clustering was based on homozygosity and heterozygosity of functionally characterized genes among all the accessions. Genetic marker data divided the accessions into four clusters based on allelic variation. First cluster represented accessions from Hong Kong (419,218), Pakistan (18,650, 32,122), India (163,309), Georgia (647,858), and France (261,789). Among these accessions, most of the variability was found among the genes involved in oxalate biosynthesis, including glycolate oxidase, malate dehydrogenase, ascorbate peroxidase, and isocitrate lyase. Cluster 2 represented the accessions with comparatively greater heterozygosity level in most of the genes. The accessions were belonging to Pakistan (30,802, 19,339), Belgium (179,593), Macedonia (379,549), China (648,942), Japan (227,230), and Iran (227,383). Cluster 3 was characterized with the accessions having comparatively less variability in oxalate-related genes and greater variability in downy mildew resistance gene. The cluster included the accessions from South Korea (508,504), Georgia (647,853), Afghanistan (212,119), China (648,944), Taiwan (321,020), and United States (184,379). Cluster 4 was the intermediate among all, consisting of most of the accessions from Pakistan (30,526, 18,649, 18,554, 18,655, 32,618, 163,310, and 30,518) and three from Germany (266,926), Mongolia (604,785), Turkey (171,860), and Syria (181,964). This group was at the borderline of homozygosity and heterozygosity. Most variability contributing genes included formate dehydrogenase, ascorbate peroxidase, citrate synthase, and ascorbate oxidase.

The cluster-based relationship revealed grouping of accessions irrespective to its geographical locations. However, the variation was found to be more linked with nutritional variation of accessions. Similar results were reported by Hu et al. (2007) in its genetic marker study. Accessions from same region were clustered into the different groups, showing its independence to observed genetic variability. The reason of this variation might be due to the introduction of similar germplasm in different areas, resulting in genetic drift or natural selection. Heat map clustering represented grouping of accessions based on presence of various genes. Accessions from Pakistan, India, Georgia, Belgium, Macedonia, China, Japan, Iran, Germany, Mongolia, and Turkey were found to be more variable in terms of disease resistance gene. Besides, the accessions from India, Pakistan, Georgia, Belgium, and China were also found to be rich in minerals, especially sodium, potassium, zinc, and manganese.

## Conclusion

It was concluded from the findings that the minerals are required not only for proper growth and development of plants, but also for making them resistant to various diseases. The accessions from India, Pakistan, Iran, and Belgium showed high variability in terms of oxalate metabolism-related genes. The accessions from Iran and Belgium were nutritionally more variable in terms of protein, ash, and fiber contents, along with minerals. This represented the correlation of good oxalate metabolism in leafy crops with mineral absorption. These findings were also supported by the report of Sun et al. (2019) about oxalate–mineral relationship. Globally, the accessions from Asia showed more diversity compared to the accessions from other continents. Similarly, the report of Kuwahara et al. (2014) about SSR marker diversity showed East Asian spinach germplasm as the most diverse compared to European and American germplasm. Genetically diverse accessions have the natural ability to bear a number of environmental stresses. Therefore, the abovementioned diverse accessions could be considered as useful genotypes in future breeding programs.

## Data Availability Statement

The original contributions presented in the study are included in the article/Supplementary Material, further inquiries can be directed to the corresponding author/s.

## Author Contributions

MR conducted the research and wrote – original draft. ZY supervised the research. AD provided the resources for research. MM and NR interpreted data for formal analysis. AA and AY reviewed the original draft. AA, MO, and HA edited and revised the manuscript. All authors contributed to the article and approved the submitted version.

## Conflict of Interest

The authors declare that the research was conducted in the absence of any commercial or financial relationships that could be construed as a potential conflict of interest.

## Publisher’s Note

All claims expressed in this article are solely those of the authors and do not necessarily represent those of their affiliated organizations, or those of the publisher, the editors and the reviewers. Any product that may be evaluated in this article, or claim that may be made by its manufacturer, is not guaranteed or endorsed by the publisher.
