# Supplementary material for: Assessment of Mineral Nutrient Efficiency in Genetically Diverse Spinach Accessions by Biochemical and Functional Marker Strategies
Source: Front Plant Sci. 2022 May 30;13:889604. doi: 10.3389/fpls.2022.889604 (PMC9189916; doi:10.3389/fpls.2022.889604)
Supplement: Supplementary file 1 [file Table_1.DOCX]

**Table S1. Functional Markers used for Genetic Characterization of Spinach Accessions along with Amplified Product Details**

| **No.** | **Primer** | **Strand** | **Sequence of Primer (5'-3')** | **Primer Size (bp)** | **Ta (°C)** | **Product Length (bp)** | **A** | **H** | **PIC** |
| --- | --- | --- | --- | --- | --- | --- | --- | --- | --- |
| 1 | MaDehy-1 | F | TCACCTCTTGGCGGATCCTA | 20 | 52 | 365 | 6 | 0.79 | 0.76 |
|  |  | R | AACCCTAATCGACGAGAGACG | 21 |  |  |  |  |  |
| 2 | MaDehy-2 | F | CTTGGCGGATCCTACGAGAC | 20 | 54 | 358 | 5 | 0.57 | 0.62 |
|  |  | R | ACCCTAATCGACGAGAGACG | 20 |  |  |  |  |  |
| 3 | MaDehy-3 | F | CGTCTCTCGTCGATTAGGGT | 20 | 54 | 923 | 11 | 0.71 | 0.77 |
|  |  | R | TGTCTTCGAGAGGAGAGGCA | 20 |  |  |  |  |  |
| 4 | MaDehy-4 | F | ACGTCTCTCGTCGATTAGGGT | 21 | 55 | 312 | 7 | 0.65 | 0.64 |
|  |  | R | TTTCGTTACGGAACCGCGTA | 20 |  |  |  |  |  |
| 5 | MaDehy-5 | F | CGTCTCTCGTCGATTAGGGTT | 21 | 55 | 403 | 6 | 0.64 | 0.7 |
|  |  | R | GGCTGACCTATACCACCAGC | 20 |  |  |  |  |  |
| 6 | MaDehy-6 | F | AACGCATCGTTTGGTTGTCG | 20 | 55 | 409 | 6 | 0.61 | 0.69 |
|  |  | R | ACCCTAATCGACGAGAGACGTA | 22 |  |  |  |  |  |
| 7 | MaDehy-7 | F | TACGTCTCTCGTCGATTAGGGT | 22 | 55 | 309 | 4 | 0.5 | 0.67 |
|  |  | R | GTTACGGAACCGCGTAGAGT | 20 |  |  |  |  |  |
| 8 | MaDehy-8 | F | GCGGATCCTACGAGACAACC | 20 | 55 | 356 | 5 | 0.51 | 0.65 |
|  |  | R | AAACCCTAATCGACGAGAGACG | 22 |  |  |  |  |  |
| 9 | MaDehy-9 | F | GGCGGATCCTACGAGACAAC | 20 | 51 | 354 | 6 | 0.52 | 0.61 |
|  |  | R | CCCTAATCGACGAGAGACGTA | 21 |  |  |  |  |  |
| 10 | MaDehy-10 | F | GAGCCCGTGGCATCGATATT | 20 | 54 | 382 | 7 | 0.58 | 0.67 |
|  |  | R | ACGAGACTCAGGTGAAATCCT | 21 |  |  |  |  |  |
| 11 | AscPerox-1 | F | CTCATGGAGCCAACAGTGGT | 20 | 51 | 912 | 20 | 0.92 | 0.92 |
|  |  | R | TGAAAGACATACAGCAGCGT | 20 |  |  |  |  |  |
| 12 | AscPerox-2 | F | AGATGGCACTCTGCTGGAAC | 20 | 55 | 999 | 15 | 0.8 | 0.85 |
|  |  | R | GGGAATGAAAGACATACAGCAGC | 23 |  |  |  |  |  |
| 13 | AscPerox-3 | F | TCCACCCTGGAAGAGAGGTT | 20 | 53 | 882 | 16 | 0.81 | 0.83 |
|  |  | R | ACCTGCAACCAACAGATACA | 20 |  |  |  |  |  |
| 14 | AscPerox-4 | F | ACGCTGCTGTATGTCTTTCAT | 21 | 54 | 250 | 7 | 0.5 | 0.61 |
|  |  | R | AGCATCAGCAAACCTGCAAC | 20 |  |  |  |  |  |
| 15 | AscPerox-5 | F | GCTGCTGTATGTCTTTCATTCCC | 23 | 55 | 141 | 6 | 0.55 | 0.67 |
|  |  | R | AAGCTTCATGTGGGCCTCAG | 20 |  |  |  |  |  |
| 16 | AscPerox-6 | F | AGGGAACTCTTGAGCGGAGA | 20 | 55 | 121 | 5 | 0.45 | 0.7 |
|  |  | R | GGAATGAAAGACATACAGCAGCG | 23 |  |  |  |  |  |
| 17 | AscPerox-7 | F | ACCCCATCAGGGAGCAATTC | 20 | 54 | 869 | 13 | 0.7 | 0.82 |
|  |  | R | AATGAAAGACATACAGCAGCGT | 22 |  |  |  |  |  |
| 18 | AscPerox-8 | F | CGCTGCTGTATGTCTTTCATTCC | 23 | 55 | 416 | 9 | 0.67 | 0.7 |
|  |  | R | GTTCAAATCGCAACCCACCG | 20 |  |  |  |  |  |
| 19 | AscPerox-9 | F | AGCTCATGGAGCCAACAGTG | 20 | 55 | 917 | 15 | 0.78 | 0.89 |
|  |  | R | GAATGAAAGACATACAGCAGCGT | 23 |  |  |  |  |  |
| 20 | AscPerox-10 | F | ACGCTGCTGTATGTCTTTCATT | 22 | 54 | 146 | 7 | 0.52 | 0.65 |
|  |  | R | AGAAAGCTTCATGTGGGCCT | 20 |  |  |  |  |  |
| 21 | GlyOx-1 | F | ATCTTGTTTCGTCCCCGCAT | 20 | 52 | 308 | 5 | 0.73 | 0.69 |
|  |  | R | GCTCTCTCGGCTCTTCTGAC | 20 |  |  |  |  |  |
| 22 | GlyOx-2 | F | CTTGCTCCTTCCTCCCAGTG | 20 | 55 | 504 | 6 | 0.71 | 0.68 |
|  |  | R | CAGCTCTCTCGGCTCTTCTG | 20 |  |  |  |  |  |
| 23 | GlyOx-3 | F | TGGAGCTGAGGATGAGTGGA | 20 | 55 | 370 | 5 | 0.55 | 0.67 |
|  |  | R | CTTGAACCCAGCTCTCTCGG | 20 |  |  |  |  |  |
| 24 | GlyOx-4 | F | AAGGCAGGAGAAAATGGGGG | 20 | 55 | 467 | 4 | 0.5 | 0.59 |
|  |  | R | CCTTGAACCCAGCTCTCTCG | 20 |  |  |  |  |  |
| 25 | GlyOx-5 | F | CAGAAGAGCCGAGAGAGCTG | 20 | 55 | 857 | 8 | 0.62 | 0.71 |
|  |  | R | GAACGACCCAGTTACGAGGG | 20 |  |  |  |  |  |
| 26 | GlyOx-6 | F | CGAGAGAGCTGGGTTCAAGG | 20 | 55 | 821 | 8 | 0.6 | 0.75 |
|  |  | R | GAGTACGTGTGTTGCTTGCG | 20 |  |  |  |  |  |
| 27 | GlyOx-7 | F | CCGAGAGAGCTGGGTTCAAG | 20 | 55 | 152 | 3 | 0.45 | 0.5 |
|  |  | R | GCTCTTGTCCATCTCTGCGA | 20 |  |  |  |  |  |
| 28 | GlyOx-8 | F | GCTTGTCAGAAGAGCCGAGA | 20 | 55 | 837 | 9 | 0.65 | 0.75 |
|  |  | R | GGAGTACGTGTGTTGCTTGC | 20 |  |  |  |  |  |
| 29 | GlyOx-9 | F | AGCTTGTCAGAAGAGCCGAG | 20 | 55 | 149 | 3 | 0.46 | 0.56 |
|  |  | R | GAGGTCCAGGCCTTCGAAAT | 20 |  |  |  |  |  |
| 30 | GlyOx-10 | F | GAGAGAGCTGGGTTCAAGGC | 20 | 55 | 755 | 10 | 0.69 | 0.78 |
|  |  | R | TTAATCGGCGTGCTCTCTGG | 20 |  |  |  |  |  |
| 31 | IsoLy-1 | F | TACCGTTGGCTTGATGGCTT | 20 | 51 | 310 | 14 | 0.91 | 0.91 |
|  |  | R | ACGACCCATAATTCTCCTTTGT | 22 |  |  |  |  |  |
| 32 | IsoLy-2 | F | GCTGAGCGAAGAAGAGGTGT | 20 | 54 | 504 | 16 | 0.8 | 0.87 |
|  |  | R | AAACGACCCATAATTCTCCTTTGT | 24 |  |  |  |  |  |
| 33 | IsoLy-3 | F | GATGTGTGAGTGGACGAGCA | 20 | 54 | 419 | 10 | 0.75 | 0.85 |
|  |  | R | ACGACCCATAATTCTCCTTTGTA | 23 |  |  |  |  |  |
| 34 | IsoLy-4 | F | CAAGTCTGCACTTCCGCAAC | 20 | 55 | 164 | 6 | 0.48 | 0.5 |
|  |  | R | AAAACGACCCATAATTCTCCTTTGT | 25 |  |  |  |  |  |
| 35 | IsoLy-5 | F | TCGTAACCAAGCACACCGAA | 20 | 54 | 714 | 13 | 0.76 | 0.82 |
|  |  | R | AGAAAACGACCCATAATTCTCCTT | 24 |  |  |  |  |  |
| 36 | IsoLy-6 | F | TTCGTAACCAAGCACACCGA | 20 | 54 | 716 | 13 | 0.72 | 0.76 |
|  |  | R | AAGAAAACGACCCATAATTCTCCT | 24 |  |  |  |  |  |
| 37 | IsoLy-7 | F | TCCACCAAGTCTGCACTTCC | 20 | 54 | 167 | 4 | 0.35 | 0.47 |
|  |  | R | AACGACCCATAATTCTCCTTTGTA | 24 |  |  |  |  |  |
| 38 | IsoLy-8 | F | TGTCATGGTACTGCTGAGCG | 20 | 54 | 519 | 7 | 0.5 | 0.61 |
|  |  | R | AGAAAACGACCCATAATTCTCCTTT | 25 |  |  |  |  |  |
| 39 | IsoLy-9 | F | GCGCCTTGTTAGCTTGAACC | 20 | 54 | 201 | 4 | 0.36 | 0.41 |
|  |  | R | AAGAAAACGACCCATAATTCTCCTT | 25 |  |  |  |  |  |
| 40 | IsoLy-10 | F | GGCGCCTTGTTAGCTTGAAC | 20 | 54 | 196 | 4 | 0.34 | 0.4 |
|  |  | R | ACGACCCATAATTCTCCTTTGTAT | 24 |  |  |  |  |  |
| 41 | MaSyn-1 | F | CCAATACCTCTGGCTCTGCAT | 21 | 52 | 145 | 8 | 0.79 | 0.77 |
|  |  | R | GAACCCAAAACAGTGCCTCG | 20 |  |  |  |  |  |
| 42 | MaSyn-2 | F | CACAGCTCCAATCACCGTCT | 20 | 54 | 539 | 10 | 0.76 | 0.78 |
|  |  | R | GGTTTTATGCAGAGCCAGAGG | 21 |  |  |  |  |  |
| 43 | MaSyn-3 | F | GCTCCGGAGTTAAGAGCGTT | 20 | 55 | 631 | 11 | 0.78 | 0.81 |
|  |  | R | AGGTTTTATGCAGAGCCAGAGG | 22 |  |  |  |  |  |
| 44 | MaSyn-4 | F | ATCCAATACCTCTGGCTCTGC | 21 | 54 | 144 | 4 | 0.45 | 0.48 |
|  |  | R | CCCAAAACAGTGCCTCGATT | 20 |  |  |  |  |  |
| 45 | MaSyn-5 | F | ATTTGGTCGTACCGTCCTCG | 20 | 55 | 357 | 6 | 0.5 | 0.5 |
|  |  | R | GGTTTTATGCAGAGCCAGAGGT | 22 |  |  |  |  |  |
| 46 | MaSyn-6 | F | GTGTGCCTCCTTGGGAAAGT | 20 | 55 | 144 | 4 | 0.46 | 0.5 |
|  |  | R | CAGAGCCAGAGGTATTGGATGA | 22 |  |  |  |  |  |
| 47 | MaSyn-7 | F | TGTGTGCCTCCTTGGGAAAG | 20 | 54 | 155 | 5 | 0.5 | 0.51 |
|  |  | R | AGGTTTTATGCAGAGCCAGAG | 21 |  |  |  |  |  |
| 48 | MaSyn-8 | F | ATAACGCCGTCTTGCCTCTC | 20 | 54 | 462 | 7 | 0.65 | 0.69 |
|  |  | R | AGAGCCAGAGGTATTGGATGA | 21 |  |  |  |  |  |
| 49 | MaSyn-9 | F | CGGACCGGTGATCTCAACTC | 20 | 55 | 591 | 7 | 0.71 | 0.78 |
|  |  | R | TAGGTTTTATGCAGAGCCAGAGG | 23 |  |  |  |  |  |
| 50 | MaSyn-10 | F | CGCATTGACATGTGTGCCTC | 20 | 54 | 155 | 4 | 0.56 | 0.57 |
|  |  | R | CAGAGCCAGAGGTATTGGATG | 21 |  |  |  |  |  |
| 51 | CitSyn-1 | F | TTGCGAATCCGATGATGGCT | 20 | 52 | 206 | 8 | 0.85 | 0.83 |
|  |  | R | GAAACACCAAAGAGCACGGT | 20 |  |  |  |  |  |
| 52 | CitSyn-2 | F | TGAAGCCAGCCAACAAGACT | 20 | 55 | 854 | 12 | 0.86 | 0.87 |
|  |  | R | AACACCAAAGAGCACGGTGTA | 21 |  |  |  |  |  |
| 53 | CitSyn-3 | F | CTTGCGAATCCGATGATGGC | 20 | 55 | 231 | 5 | 0.45 | 0.47 |
|  |  | R | TGAGAGCAGATGCCAAGACTC | 21 |  |  |  |  |  |
| 54 | CitSyn-4 | F | GGCATCTGCTCTCAGGTAACA | 21 | 55 | 251 | 5 | 0.5 | 0.56 |
|  |  | R | AAACACGAGAGAGTGCGACA | 20 |  |  |  |  |  |
| 55 | CitSyn-5 | F | AGCCAGCCAACAAGACTGAT | 20 | 55 | 881 | 10 | 0.65 | 0.66 |
|  |  | R | TACCTGAGAGCAGATGCCAAG | 21 |  |  |  |  |  |
| 56 | CitSyn-6 | F | GCCAGCCAACAAGACTGATG | 20 | 55 | 848 | 9 | 0.7 | 0.75 |
|  |  | R | CACCAAAGAGCACGGTGTAGT | 21 |  |  |  |  |  |
| 57 | CitSyn-7 | F | AGACTGATGGAGCATGCGAA | 20 | 55 | 838 | 9 | 0.72 | 0.76 |
|  |  | R | ACACCAAAGAGCACGGTGTAG | 21 |  |  |  |  |  |
| 58 | CitSyn-8 | F | GAAGCCAGCCAACAAGACTG | 20 | 54 | 868 | 13 | 0.86 | 0.87 |
|  |  | R | GCCAAGACTCCTTGAAACACC | 21 |  |  |  |  |  |
| 59 | CitSyn-9 | F | CTTGGCATCTGCTCTCAGGTA | 21 | 55 | 232 | 5 | 0.42 | 0.46 |
|  |  | R | TCAGGACTCGGTGGTGACT | 19 |  |  |  |  |  |
| 60 | CitSyn-10 | F | CCACTTTTGAAGCCAGCCAA | 20 | 54 | 893 | 10 | 0.75 | 0.78 |
|  |  | R | GTTACCTGAGAGCAGATGCC | 20 |  |  |  |  |  |
| 61 | AscOx-1 | F | TTCCAAGCATGTCTTGTCCCA | 21 | 52 | 822 | 10 | 0.85 | 0.84 |
|  |  | R | TGCTCCGGTCAAAGTCATCC | 20 |  |  |  |  |  |
| 62 | AscOx-2 | F | TTGGGGATTCCAAGCATGTC | 20 | 54 | 750 | 5 | 0.45 | 0.46 |
|  |  | R | GTTGAGGAGGGTCAATCCCG | 20 |  |  |  |  |  |
| 63 | AscOx-3 | F | TTCCAAGCATGTCTTGTCCC | 20 | 54 | 994 | 4 | 0.5 | 0.5 |
|  |  | R | TTCATGGCACCGAGATACGG | 20 |  |  |  |  |  |
| 64 | AscOx-4 | F | CCAAATTGGGGATTCCAAGCA | 21 | 55 | 955 | 5 | 0.49 | 0.5 |
|  |  | R | ATGGCCCACTTGACATAGCC | 20 |  |  |  |  |  |
| 65 | AscOx-5 | F | TGGGAATGCAAAGATCGGCA | 20 | 54 | 982 | 6 | 0.5 | 0.55 |
|  |  | R | GGGACAAGACATGCTTGGAA | 20 |  |  |  |  |  |
| 66 | AscOx-6 | F | CCAAGCATGTCTTGTCCCAAT | 21 | 54 | 646 | 3 | 0.4 | 0.47 |
|  |  | R | TGAGGACGGAGTAGGACTCG | 20 |  |  |  |  |  |
| 67 | AscOx-7 | F | TCCAAGCATGTCTTGTCCCAAT | 22 | 55 | 854 | 7 | 0.56 | 0.6 |
|  |  | R | CCATAGCGGCGGTGATTCTA | 20 |  |  |  |  |  |
| 68 | AscOx-8 | F | TGCAAAGATCGGCAGGGTTA | 20 | 55 | 977 | 6 | 0.5 | 0.52 |
|  |  | R | TGGGACAAGACATGCTTGGAAT | 22 |  |  |  |  |  |
| 69 | AscOx-9 | F | GAATGCAAAGATCGGCAGGG | 20 | 54 | 972 | 8 | 0.67 | 0.7 |
|  |  | R | GACATGCTTGGAATCCCCAAT | 21 |  |  |  |  |  |
| 70 | AscOx-10 | F | ATTGGGGATTCCAAGCATGTC | 21 | 55 | 826 | 5 | 0.56 | 0.6 |
|  |  | R | CCGGTCAAAGTCATCCCAGG | 20 |  |  |  |  |  |
| 71 | OxSyn-1 | F | ACGGCGGAGGAATTTGAGTT | 20 | 52 | 239 | 21 | 0.92 | 0.91 |
|  |  | R | GTGTGGAGGAAGAGAGCACC | 20 |  |  |  |  |  |
| 72 | OxSyn-2 | F | TTGACTCAGCGACGGAACTC | 20 | 55 | 74 | 15 | 0.85 | 0.84 |
|  |  | R | GCTCGTAGTGCCAGAAGTGT | 20 |  |  |  |  |  |
| 73 | OxSyn-3 | F | ACACTTCTGGCACTACGAGC | 20 | 55 | 829 | 16 | 0.84 | 0.88 |
|  |  | R | GGAACACCGAATGCAACACC | 20 |  |  |  |  |  |
| 74 | OxSyn-4 | F | TTCCTCCACACTTCTGGCAC | 20 | 55 | 572 | 10 | 0.63 | 0.67 |
|  |  | R | GGCTCTTGGATCTCGCCTTT | 20 |  |  |  |  |  |
| 75 | OxSyn-5 | F | CTTGTCTCCGACGCTGGAAT | 20 | 50 | 380 | 11 | 0.62 | 0.7 |
|  |  | R | GTGCCAGAAGTGTGGAGGAA | 20 |  |  |  |  |  |
| 76 | OxSyn-6 | F | GGGTGTTGCATTCGGTGTTC | 20 | 50 | 198 | 10 | 0.65 | 0.64 |
|  |  | R | CGGCGCTGAATCTTACCAGA | 20 |  |  |  |  |  |
| 77 | OxSyn-7 | F | CGATGTCGTTGCTCTCACCT | 20 | 55 | 308 | 12 | 0.64 | 0.67 |
|  |  | R | TAACGAGTTCCGTCGCTGAG | 20 |  |  |  |  |  |
| 78 | OxSyn-8 | F | CTCAGCGACGGAACTCGTTA | 20 | 55 | 103 | 12 | 0.59 | 0.63 |
|  |  | R | ATTGAGCTGCGTAAGCGGTA | 20 |  |  |  |  |  |
| 79 | OxSyn-9 | F | CCTTCCCTAACACCGTCGAG | 20 | 55 | 303 | 11 | 0.6 | 0.62 |
|  |  | R | CGTCCGGGTGATTAACGAGT | 20 |  |  |  |  |  |
| 80 | OxSyn-10 | F | TCGTTAATCACCCGGACGAC | 20 | 55 | 316 | 9 | 0.61 | 0.65 |
|  |  | R | GTCGGCACAGCAGTATACCA | 20 |  |  |  |  |  |
| 81 | FormDeh-1 | F | TGTTTTCCTTGAACCGTTACACA | 23 | 51 | 245 | 18 | 0.88 | 0.87 |
|  |  | R | TGAGGAGCAAGTTCACCGTC | 20 |  |  |  |  |  |
| 82 | FormDeh-2 | F | AGACGATAGGAACCGTGGGA | 20 | 54 | 568 | 8 | 0.67 | 0.72 |
|  |  | R | TGTGTAACGGTTCAAGGAAAACA | 23 |  |  |  |  |  |
| 83 | FormDeh-3 | F | TGCTAACGAATGAAAAGGTGTTGT | 24 | 55 | 192 | 5 | 0.43 | 0.5 |
|  |  | R | CTGAGGAGCAAGTTCACCGT | 20 |  |  |  |  |  |
| 84 | FormDeh-4 | F | TCATGGAGAGGCAAGCAGTG | 20 | 55 | 249 | 5 | 0.48 | 0.48 |
|  |  | R | ACAACACCTTTTCATTCGTTAGCA | 24 |  |  |  |  |  |
| 85 | FormDeh-5 | F | ACACATTGGAGGGTACAGCG | 20 | 54 | 153 | 4 | 0.39 | 0.42 |
|  |  | R | GTGTAACGGTTCAAGGAAAACA | 22 |  |  |  |  |  |
| 86 | FormDeh-6 | F | GTTTTCCTTGAACCGTTACACA | 22 | 54 | 419 | 7 | 0.57 | 0.68 |
|  |  | R | AGGGGAAGTGCCCTCCTATT | 20 |  |  |  |  |  |
| 87 | FormDeh-7 | F | CATGCGCAACTTCGTACCAG | 20 | 54 | 660 | 10 | 0.78 | 0.81 |
|  |  | R | TGTGTAACGGTTCAAGGAAAAC | 22 |  |  |  |  |  |
| 88 | FormDeh-8 | F | GATAGGAACCGTGGGAGCTG | 20 | 55 | 565 | 11 | 0.76 | 0.82 |
|  |  | R | ATGTGTAACGGTTCAAGGAAAACA | 24 |  |  |  |  |  |
| 89 | FormDeh-9 | F | GCTGGTATTGGCTCGGATCA | 20 | 54 | 825 | 16 | 0.85 | 0.86 |
|  |  | R | TCGTTAGCAAAAGTTTCCAGAGA | 23 |  |  |  |  |  |
| 90 | FormDeh-10 | F | TGTTTTCCTTGAACCGTTACACAT | 24 | 55 | 307 | 6 | 0.43 | 0.47 |
|  |  | R | TGAACGTTGGGGTCGTTTCT | 20 |  |  |  |  |  |
| 91 | OxLig-1 | F | TAGGCTCCGTTTGGTTTGGT | 20 | 52 | 936 | 8 | 0.82 | 0.8 |
|  |  | R | AAGTGTTCCGCCACGATTCT | 20 |  |  |  |  |  |
| 92 | OxLig-2 | F | CTTTCGGGGTCCCTGATGAC | 20 | 55 | 92 | 4 | 0.43 | 0.51 |
|  |  | R | ACCAAACCAAACGGAGCCTA | 20 |  |  |  |  |  |
| 93 | OxLig-3 | F | TTCCAGGGGCAGAAGGAGTA | 20 | 55 | 502 | 6 | 0.65 | 0.69 |
|  |  | R | ACCAAACGGAGCCTAAAGCC | 20 |  |  |  |  |  |
| 94 | OxLig-4 | F | GTGTTAGGCTTTAGGCTCCGT | 21 | 55 | 829 | 10 | 0.52 | 0.54 |
|  |  | R | TTACCGCTTCCTCGTCAACC | 20 |  |  |  |  |  |
| 95 | OxLig-5 | F | TGTGTTAGGCTTTAGGCTCCG | 21 | 55 | 918 | 13 | 0.71 | 0.78 |
|  |  | R | TTCCCGCTTGCAGTTTTTGG | 20 |  |  |  |  |  |
| 96 | OxLig-6 | F | GCTTTTGTCGCATCCTGACC | 20 | 55 | 121 | 4 | 0.35 | 0.4 |
|  |  | R | CCAAACGGAGCCTAAAGCCTA | 21 |  |  |  |  |  |
| 97 | OxLig-7 | F | TCGTCCGTAGGGTCTATGGG | 20 | 55 | 778 | 11 | 0.72 | 0.87 |
|  |  | R | ACGGAGCCTAAAGCCTAACAC | 21 |  |  |  |  |  |
| 98 | OxLig-8 | F | TGGTCAGCAACTGAACCTCG | 20 | 55 | 360 | 8 | 0.47 | 0.54 |
|  |  | R | ACCAAACCAAACGGAGCCTAA | 21 |  |  |  |  |  |
| 99 | OxLig-9 | F | GGTCAGCAACTGAACCTCGT | 20 | 55 | 359 | 8 | 0.45 | 0.54 |
|  |  | R | ACCAAACCAAACGGAGCCT | 19 |  |  |  |  |  |
| 100 | OxLig-10 | F | ATCTTCCAGGGGCAGAAGGA | 20 | 55 | 506 | 9 | 0.51 | 0.53 |
|  |  | R | AACCAAACGGAGCCTAAAGC | 20 |  |  |  |  |  |
| 101 | G6PDH-1 | F | GGGATTCAGTGCCGAAGCTA | 20 | 51 | 201 | 11 | 0.87 | 0.86 |
|  |  | R | TCTTGGCACTTGTCATCGTTC | 21 |  |  |  |  |  |
| 102 | G6PDH-2 | F | AGAACGATGACAAGTGCCAAG | 21 | 55 | 73 | 9 | 0.73 | 0.78 |
|  |  | R | TGGGTGTTTCTGTGTTGTGGT | 21 |  |  |  |  |  |
| 103 | G6PDH-3 | F | AGTGACCCAGTTTCAGGCAC | 20 | 55 | 243 | 7 | 0.64 | 0.75 |
|  |  | R | CTTGGCACTTGTCATCGTTCT | 21 |  |  |  |  |  |
| 104 | G6PDH-4 | F | CTAAGGGGGATTCAGTGCCG | 20 | 55 | 207 | 8 | 0.69 | 0.79 |
|  |  | R | TCTTGGCACTTGTCATCGTTCT | 22 |  |  |  |  |  |
| 105 | G6PDH-5 | F | TGCTAAGGGGGATTCAGTGC | 20 | 54 | 207 | 5 | 0.51 | 0.67 |
|  |  | R | TTGGCACTTGTCATCGTTCT | 20 |  |  |  |  |  |
| 106 | G6PDH-6 | F | TAGCAAGATCCCCAGAAGCG | 20 | 55 | 283 | 7 | 0.71 | 0.78 |
|  |  | R | TTCTTGGCACTTGTCATCGTTC | 22 |  |  |  |  |  |
| 107 | G6PDH-7 | F | GCGCCAAGCACAACAATAGA | 20 | 53 | 264 | 5 | 0.5 | 0.68 |
|  |  | R | CTTGGCACTTGTCATCGTTC | 20 |  |  |  |  |  |
| 108 | G6PDH-8 | F | GAACGATGACAAGTGCCAAGA | 21 | 54 | 469 | 10 | 0.75 | 0.79 |
|  |  | R | TGCATGTGTTGTGAAACACCC | 21 |  |  |  |  |  |
| 109 | G6PDH-9 | F | AGAACGATGACAAGTGCCAAGA | 22 | 54 | 164 | 6 | 0.61 | 0.67 |
|  |  | R | GGACTGTGCACACAATTCCA | 20 |  |  |  |  |  |
| 110 | G6PDH-10 | F | CACGCAACCGGTTTCTCAAT | 20 | 55 | 727 | 4 | 0.57 | 0.66 |
|  |  | R | TCTTGGCACTTGTCATCGTTCTA | 23 |  |  |  |  |  |

**Key:** Ta = Annealing temperature, A = Alleles, H = Heterozygosity, PIC = Polymorphism information content
